# Energy cost of walking in obese survivors of acute lymphoblastic leukemia: A report from the St. Jude Lifetime Cohort

**DOI:** 10.3389/fped.2022.976012

**Published:** 2022-10-28

**Authors:** Matthew D. Wogksch, Emily R. Finch, Vikki G. Nolan, Matthew P. Smeltzer, Fawaz Mzayek, Chelsea G. Goodenough, Ching-Hon Pui, Hiroto Inaba, Daniel A. Mulrooney, Sue C. Kaste, Tara M. Brinkman, Jennifer Q. Lanctot, Deo Kumar Srivastava, John L. Jefferies, Gregory T. Armstrong, Leslie L. Robison, Melissa M. Hudson, Kirsten K. Ness

**Affiliations:** ^1^ Department of Epidemiology and Cancer Control, St. Jude Children’s Research Hospital, Memphis, TN, United States; ^2^ Division of Epidemiology, Biostatistics, and Environmental Health, School of Public Health, University of Memphis, Memphis, TN, United States; ^3^ Department of Oncology, St. Jude Children’s Research Hospital, Memphis, TN, United States; ^4^ Department of Diagnostic Imaging, St. Jude Children’s Research Hospital, Memphis, TN, United States; ^5^ Department of Radiology, University of Tennessee Health Science Center, Memphis, TN, United States; ^6^ Department of Psychology, St. Jude Children’s Research Hospital, Memphis, TN, United States; ^7^ Department of Biostatistics, St. Jude Children’s Research Hospital, Memphis, TN, United States; ^8^ Division of Cardiovascular Diseases, Institute for Cardiovascular Science, University of Tennessee Health Science Center, Memphis, TN, United States

**Keywords:** childhood cancer, fitness, obesity, quality of life, acute lymphoblastic leukemia

## Abstract

**Purpose:**

Adult survivors of childhood acute lymphoblastic leukemia (ALL) have impaired adaptive physical function and poor health-related quality of life (HRQoL). Obesity may contribute to these impairments by increasing the physiological cost of walking. Due to treatment exposures during ALL therapy, survivors’ cost of walking may be more impacted by obesity than the general population. Therefore, we examined associations between obesity, persistent motor neuropathy, and energy cost of walking; and examined associations between energy cost of walking, adaptive physical function, and HRQoL, in adult survivors of childhood ALL vs. community controls.

**Methods:**

Obesity was measured *via* body mass index (BMI) and body fat percentage. The physiological cost index (PCI) was calculated from the six-minute walk test. Adaptive physical functioning was measured using two tests: the timed up and go (TUG) test and the physical performance test. Persistent motor neuropathy was measured using the modified total neuropathy score; HRQoL was measured using the Short-Form-36 questionnaire. The associations between obesity and PCI were evaluated using multivariable linear regressions in adult survivors of childhood ALL (*n* = 1,166) and community controls (*n* = 491). Then, the associations between PCI, adaptive physical functioning and peripheral neuropathy were examined using multivariable linear regressions. Finally, to determine the association between obesity, and neuropathy on PCI, while accounting for potential lifestyle and treatment confounders, a three model, sequential linear regression was used.

**Results:**

Obese individuals (BMI > 40 kg/m^2^ and excess body fat percentage [males: >25%; females: >33%]) had higher PCI compared to those with normal BMI and body fat percentage (0.56 ± 0.01 vs. 0.49 ± 0.009 beats/meter *p *< .01; and 0.51 ± 0.007 vs. 0.48 ± .0006 beats/meter *p *< .01, respectively). Treatment exposures did not attenuate this association. Increased PCI was associated with longer TUG time in survivors, but not community controls (6.14 ± 0.02 s vs. 5.19 ± 0.03 s, *p *< .01). Survivors with PCI impairment >95th percentile of community controls had lower HRQoL compared to un-impaired ALL survivors: 46.9 ± 0.56 vs. 50.4 ± 1.08, respectively (*p *< .01).

**Conclusion:**

Obesity was associated with increased PCI. Survivors with high PCI had disproportionately worse adaptive physical function and HRQoL compared to controls. Survivors with increased energy costs of walking may benefit from weight loss interventions.

## Introduction

Due to advances in cancer therapy and supportive care, the 5-year survival rate of childhood acute lymphoblastic leukemia (ALL) now exceeds 90%, with ALL representing 19% of all long-term childhood cancer survivors (1). Over the past six decades, therapy for ALL has not only improved survival, but has also been refined to minimize long-term morbidities and optimize health for survivors (2). Nevertheless, survivors of childhood ALL, when compared to their peers, remain at increased risk for chronic health conditions, including obesity, that interfere with activities of daily living (3).

Obesity is a prevalent public health problem associated with chronic disease, including metabolic disease, cardiovascular disease, secondary cancers, and type II diabetes (4–6). It is particularly concerning in ALL survivors, as past treatment exposures confer risk for comorbidities, including diabetes and cardiovascular disease. Obesity perpetuates these chronic health conditions. Although obesity may be a modifiable condition, it may be difficult to manage in adult survivors of childhood ALL, particularly in ALL survivors with neuromusculoskeletal problems (i.e., persistent motor neuropathy) that interfere with health optimizing behaviors, including engagement in physical activity (PA) (7). Additionally, ALL survivors have increased sedentary behavior compared to peers, which may independently increase risk for these chronic health conditions (8).

Energy cost is the relative oxygen cost required to perform an activity or to move the body through space (9). In the general population, adults with obesity have a higher energy cost of performing activities compared to those without obesity (10). Higher energy cost of activity changes the perception of exertion while moving, increases fatiguability, and reduces time spent in PA (11). This results in a reciprocal association between PA and obesity: obesity contributes to reduced PA because movement is difficult; less movement reduces overall energy expenditure and, if not countered by reduced energy intake, results in weight gain (12, 13). Excess body weight taxes the body’s systems. Initially, among overweight persons, only vigorous activity may seem difficult. however, this impairment may interfere if job demands, or leisure activities, require vigorous organ system responses. Subsequently, as body weight increases, less intensive activities, e.g., walking, housework, grocery shopping, or getting out of a chair, become difficult, limiting participation in important life roles, and eventually interfering with quality of life (14).

When compared to peers, adult survivors of childhood ALL spend significantly less time in moderate to vigorous PA (15). They are also at an increased risk for difficulties with performing activities of daily living that require negotiating the environment outside of the home, and have a lower physical component summary score (PCS), a measure of physical health-related quality of life (HRQoL) (16). Peripheral neuropathy is a prevalent outcome in ALL survivors, and is related to cancer therapy (13). Associations between peripheral neuropathy, activities of daily living, and HRQoL are well known (17–20). However, the impact of obesity, and peripheral neuropathy, on the energy costs of daily activity, adaptive physical function, and HRQoL, have not been explored in this population. Understanding these associations have the potential to provide information to help tailor interventions designed to increase PA and/or promote weight loss, which have previously produced inconsistent results (21). Thus, the aims of this study were to examine associations between obesity, persistent motor neuropathy and energy cost of walking, and to examine associations between energy cost of walking, adaptive physical function, and HRQoL in adult survivors of childhood ALL.

## Materials and methods

### Study population

Participants included members of the St. Jude Lifetime Cohort (22), assembled to assess health outcomes in aging survivors of childhood malignancies. For these analyses, we included any adult survivors (≥18 years of age) of childhood ALL treated at St. Jude Children’s Research Hospital (since it’s opening) that were at least 10 years from their original diagnosis (1962–2012). Participants also must have completed an on-campus assessment [e.g., self-reported questionnaires and a functional assessment (*n* = 1,166, age range 18.0–65.5 years)]. A comparison group (community controls) was also recruited from parents/relatives of current pediatric patients, and adult friends of survivors. Controls were not first-degree relatives of St. Jude Lifetime Cohort participants, had no history of childhood cancer, but did not have to be free of other chronic disease (*n* = 491, age range 18.2–70.2 years). The St. Jude Children’s Research Hospital Institutional Review Board approved all procedures. Written informed consent was obtained on all participants prior to testing.

### Anthropometrics

Height in centimeters (cm) and weight in kilograms (kg) were measured using a wall-mounted stadiometer and electronic scale (Model 5002, ScaleTronix, Inc., Wheaton, IL). Body mass index (BMI) was calculated and categorized as underweight (<18.5 kg/m^2^), normal weight (18.5–24.9 kg/m^2^), overweight (25.0–29.9 kg/m^2^), obese grade I (30.0–34.9 kg/m^2^), obese grade II (35.0–39.9 kg/m^2^), and obese grade III (≥40.0 kg/m^2^). Percent body fat was measured *via* the three site technique (males: pectoral, abdominal, and thigh; females: triceps, suprailiac, and thigh) by trained exercise physiologists, using the Harpenden skinfold caliper (Baty, West Sussex, United Kingdom) (23). The three site skinfold technique is a valid alternative to dual energy x-ray absorptiometry in childhood cancer survivors (24). Survivors and controls were categorized as having normal body fat (males: <27.5%; females: <39.9%) or excess body fat (males: ≥27.5%; females: ≥39.9%) (25).

### Outcomes

To measure the energy cost of walking, the physiological cost index (PCI) was calculated (maximal heart rate while walking—resting heart rate)/(meters walked) (beats per meter) from the six-minute walk test (6 MWT). While the PCI calculation from the 6 MWT is not been specifically validated in adult survivors of childhood cancer, it is a valid measure of walking efficiency in children with cystic fibrosis (26), persons with traumatic brain injury (27) or stroke (28), non-disabled older adults (29), persons with amputation (30), and survivors of polio (31). Before the test, participants were asked to sit quietly for five minutes to acquire resting heart rate. Participants were then instructed to walk as fast as they could around a 41-meter track. Heart rate (measured using the Masimo Rad-5 handheld pulse oximeter, Irvine, CA) was taken at two-, four-, and six-minutes. To meet the assumption that the participants’ heart rate was in steady state, maximal heart rate was defined as the highest heart rate at the four-minute or six-minute measurement.

To assess HRQoL, participants completed the Medical Outcomes Survey Short-Form-36 (SF-36), which has been previously validated in adult survivors of childhood cancer (32). The SF-36 contains eight subscales: physical function, role physical, vitality, bodily pain, general health, social functioning, role emotional and mental health; and two summary scores: PCS and mental component summary (MCS). For these analyses, we included subscale scores and summary scores. Raw scores were calculated and converted into T-scores: with the general population mean 50 and standard deviation 10. Higher scores indicated better HRQoL.

Adaptive physical functioning was assessed using a 7-item physical performance test (PPT) and a timed up-and-go (TUG) test. The PPT is an examination of fine and gross motor skills designed to simulate activities of daily living. More specifically, the examiner evaluates the participant on how fast they can write a sentence, simulate eating by moving five beans into an empty bowl with a spoon, lift an object onto a shelf, put on and remove a jacket, pick up a coin from the floor, and turn 360 degrees and walk 50 feet. The PPT is more sensitive than traditional self-reports in detecting loss of function (33). The TUG test evaluates balance and mobility, measured as the time required to stand up from a seated position, walk three meters, turn around, walk back three meters, and sit down again. The TUG has excellent reliability (intraclass correlation coefficient: 0.93) and good discriminate validity (area under the curve: 0.65) (34).

### Other measures

Diagnosis and treatment information were obtained from medical records by trained abstractors and included type of individual chemotherapeutic agents and if survivors received cranial radiation therapy. Demographic information such as smoking status and educational attainment were obtained from questionnaires that the participants complete during their St. Jude Lifetime Cohort evaluation.

Physical activity levels were determined using six self-report items from the National Health and Nutrition Examination Survey (35). The questions asked if the participant spent at least ten minutes doing vigorous PA, on how many days per week they did these activities, and for how many total minutes per day; identical questions were asked for moderate physical activities. Weekly minutes of vigorous activity were multiplied by six; weekly minutes of moderate activities were multiplied by three and summed to get metabolic equivalent minutes per week. For analysis, PA was dichotomized into meeting (≥450 metabolic equivalent minutes per week) or not meeting the 2018 Centers for Disease Control and Prevention (CDC) guidelines (36).

Peripheral nervous system integrity was evaluated with the modified Total Neuropathy Scale (37) consisting of self-reported sensory and motor symptoms, and quantitative testing. Protective sensation was evaluated with a 4.17 log force Semmes Weinstein Monofilament. Vibration was measured using a Bioesthesiometer with a threshold of 9.0 volts (Bio-Medical Instrument Company, Newbury, OH). Manual muscle testing was performed on the fingers, ankles, and wrists (37). Reflex testing was performed in the ankles, knees, brachioradialis, biceps, and triceps tendons. A total score of 24 was possible: having fewer or no symptoms or measured deficits equated to having lower scores (37).

### Statistical analysis

Descriptive statistics of participants vs. non-participants characteristics and survivors vs. community controls were calculated and compared using chi-squared tests or Fischer’s exact test for categorical variables, and two sample t-tests for continuous variables. Multivariable linear regressions were used to examine the associations of BMI and percent body fat with PCI in survivors and community controls. The models were adjusted for potential confounders (age, sex, race, and smoking status) that changed the beta estimate for the association between BMI or body fat percentage and PCI by more than ten percent. Initial models included interaction terms (BMI × group and percent body fat × group) which were not significant. Due to the changing trends in PA over the decades, as well as the marked increase in 5-year survival starting in the 1980s for ALL, a supplemental analysis limited survivors to those treated after 1980 to see if results differed when those treated in the 1960s and 1970s were not included (38, 39). To determine the effects of neuropathy and obesity on the energy cost of walking among survivors, while accounting for potential confounders of these associations, three sequential regression models were used (40). The first model regressed past treatment exposures and prevalent neuropathy on PCI; the second regressed past treatment exposures, BMI, and prevalent neuropathy on PCI, and the third model regressed treatment exposures, BMI, prevalent neuropathy, and lifestyle factors on PCI (40). Multivariable linear models, adjusted for potential confounders (age, sex, race, smoking status, and PA), and that included a PCI by group (survivor vs. control) interaction term were used to determine associations between PCI (as both a continuous measure and as two separate dichotomies (>90th percentile (impaired) vs. ≤90th percentile (not impaired); >95th percentile (severely impaired) vs. ≤95th percentile (not severely impaired), adaptive physical functioning and HRQoL. Variables that modified associations between PCI and either adaptive physical functioning or HRQoL were retained (41). Data were analyzed with SAS version 9.4 (SAS Institute, Cary, NC).

## Results

### Characteristics of participants

Among 1,521 adult ALL survivors potentially eligible for analysis, 1,166 (76.7%) participants completed an on campus visit and a functional assessment that included the 6 WMT, while the remaining 355 eligible survivors refused to participate, could not be contacted, completed a survey only, or did not complete the 6 MWT (Supplementary Figure S1). Compared to non-participants, participants were older, more likely to have received glucocorticoids, 6-mercaptopurine, cytarabine, doxorubicin, daunorubicin, and etoposide, but less likely to have received cranial irradiation (Supplementary Table S1).

Compared to community controls, survivors were older and more likely to be male, more likely to report current smoking, and less likely to report adequate PA, and had lower educational attainment. Survivors were also more likely to be categorized as obese grade I, obese grade II, and were more likely to have excess body fat vs. community controls (Table 1).

**Table 1:** Demographic characteristics of acute lymphoblastic leukemia survivors and the community controls.

	Survivors (*n* = 1,166)	Controls (*n* = 491)	*p*
Age at evaluation in years, mean (SD)	37.3 (9.9)	34.8 (10.1)	0.001
Age at diagnosis in years, mean (SD)	6.8 (4.5)	N/A N/A	N/A
Sex, *n* (%)			
Male	607 (52.0)	221 (44.5)	0.001
Female	559 (48.0)	275 (55.4)	
Race,^a^ *n* (%)			
White	1,036 (88.8)	432 (88.0)	0.15
Black	104 (8.9)	40 (8.1)	
Other	26 (2.2)	19 (3.9)	
Body mass index, *n* (%)			
Normal weight	292 (25.1)	174 (35.4)	0.001
Overweight	323 (27.7)	133 (27.1)	
Obese grade I	266 (22.8)	77 (15.7)	
Obese grade II	153 (13.1)	52 (13.1)	
Obese grade III	111 (9.5)	43 (8.8)	
Underweight	21 (1.8)	12 (2.4)	
Body fat percentage, *n* (%)			
Normal	518 (44.4)	336 (67.7)	0.001
Excess	648 (55.6)	160 (32.3)	
Smoking status, *n* (%)			
Past	130 (11.2)	73 (14.7)	0.04
Current	229 (19.6)	79 (15.9)	
Never	807 (69.3)	344 (69.3)	
Met physical activity guidelines, *n* (%)			
Yes	571 (49.0)	297 (60.0)	0.001
No	595 (51.0)	199 (40.0)	
College degree, *n* (%)			
Yes	417 (35.8)	271 (54.6)	0.001
No	733 (62.9)	217 (43.8)	
Not reported	16 (1.3)	8 (1.6)	

Body mass index (BMI) classifications: normal weight (BMI 18.5–24.9 kg/m^2^); overweight (BMI 25.0–29.9 kg/m^2^); obese grade I (BMI 30.0–34.9 kg/m^2^); obese II (BMI 35.0–39.9 kg/m^2^); obese III (BMI ≥ 40.0 kg/m^2^); underweight (BMI < 18.5 kg/m^2^).

Normal body fat percentage (males: <27.5%; females: <39.9%); excess body fat percentage (males: ≥27.5%; females: ≥39.9%).

Physical activity guidelines based on the 2018 CDC recommendations of 450 metabolic equivalent minutes per week.

BMI, body mass index; CDC, Centers for Disease Control and Prevention; kg/m^2^, kilogram per square meter; <, less than; ≥, more than or equal to; n, number; %, percent; *p*, probability; SD, standard deviation; N/A, not applicable.

^a^ Fisher exact test used for analysis.

### Physiological cost index

Means (±SE) of the PCI in survivors and community controls, categorized by BMI and body fat percentage, are shown in Table 2. After removing nine survivors and seven community controls who could not complete the 6 MWT (see Table 2 footnote for reasons), the PCI was significantly higher in individuals classified by BMI with grade III obesity compared to those with normal weight; and those with excess body fat percentage, compared to those who were normal. The PCI was also significantly higher in survivors compared to community controls in the model with BMI as an independent predictor of PCI, but not in the model when body fat percentage was used as the independent variable. There was no interaction between survivor status and either BMI or body fat percentage; interaction terms were not included in final models. Limiting the models to survivors treated after 1980 did not significantly change the results (Supplementary Table S2).

**Table 2:** Associations between physiological cost index and body mass index or body fat percentage in adult survivors of childhood acute lymphoblastic leukemia vs. community controls.

	*n*	Physiological cost index (BMI model)			Physiological cost index (Body fat percentage model)		
		mean	SE	*p*	mean	SE	*p*
Group							
Community control	484	0.49	0.009	REF	0.50	0.008	REF
ALL survivors	1,157	0.51	0.008	0.03	0.51	0.006	0.06
Body mass index							
Normal weight	462	0.49	0.008	REF	N/A	N/A	N/A
Overweight	454	0.49	0.008	0.62	N/A	N/A	N/A
Obese grade I	340	0.49	0.009	0.43	N/A	N/A	N/A
Obese grade II	203	0.50	0.01	0.28	N/A	N/A	N/A
Obese grade III	150	0.55	0.01	<0.01	N/A	N/A	N/a
Underweight	32	0.45	0.03	0.32	N/A	N/A	N/A
Body fat percentage							
Normal	843	N/A	N/A	N/A	0.48	0.006	REF
Excess	798	N/A	N/A	N/A	0.51	0.007	<0.01

Body mass index (BMI) classifications: normal weight (BMI 18.5–24.9 kg/m^2^); overweight (BMI 25.0–29.9 kg/m^2^); obese grade I (BMI 30.0–34.9 kg/m^2^); obese II (BMI 35.0–39.9 kg/m^2^); obese III (BMI ≥ 40.0 kg/m^2^); underweight (BMI < 18.5 kg/m^2^).

Normal body fat percentage (males: <27.5%; females: <39.9%); excess body fat percentage (males: ≥27.5%; females: ≥39.9%).

Models were adjusted for sex, age at evaluation, physical activity, height, and smoking status.

Nine survivors did not complete the six-minute walk and were removed from analysis due to uncontrolled asthma (1), paralysis (2), painful swelling in legs (2), acute neurological symptoms (1), chronic low back pain (2), and a recent triple bypass surgery (1).

Seven community controls were removed from analysis due to a recently placed pacemaker (1), acute angina (1), technical error (2), severe COPD (1), unable to ambulate without assistance (1), and severe shortness of breath (1).

BMI, body mass index; <, less than; n, number; N/A, not applicable; %, percent; *p*, probability; SE, standard error; REF, reference.

### Treatment exposures, persistent motor neuropathy, body composition, and physiological cost index

Table 3 summarizes associations between persistent motor neuropathy, and PCI in three sequential multivariable models (A, B, and C) in survivors. After adjustment for treatment exposures age at analysis, age at diagnosis, and sex, survivors with persistent motor neuropathy had a higher energy cost of walking than those without persistent motor neuropathy (0.57 ± 0.04 vs. 0.52 ± 0.03 beats/meter) (Model A). With additional adjustment for the BMI categories, the association between persistent motor neuropathy and PCI was attenuated (Model B). After adjustment for all variables in models A and B and for lifestyle factors (smoking, PA), both grade III obesity and neuropathy were associated with the PCI (Model C).

**Table 3:** Sequential linear regressions examining associations between treatment exposures, BMI, prevalent neuropathy, and lifestyle factors on physiological cost index in adult survivors of childhood acute lymphoblastic leukemia.

	*n*	Physiological cost index								
		Model A (R^2^ = 0.08)			Model B (R^2^ = 0.10)			Model C (R^2^ = 0.11)^a^		
		mean	SE	*p*	mean	SE	*p*	mean	SE	*p*
Cranial Radiation										
Yes	549	0.54	0.04	0.73	0.53	0.04	0.19	0.53	0.03	0.21
No	608	0.55	0.03	REF	0.54	0.03	REF	0.51	0.04	REF
Anthracyclines										
Yes	890	0.56	0.03	0.08	0.55	0.03	0.05	0.53	0.04	0.07
No	267	0.53	0.03	REF	0.52	0.03	REF	0.51	0.04	REF
Corticosteroids										
Yes	1,150	0.53	0.01	0.64	0.52	0.01	0.64	0.51	0.01	0.78
No	7	0.55	0.06	REF	0.55	0.07	REF	0.53	0.06	REF
Neuropathy										
Yes	89	0.57	0.04	0.03	0.55	0.04	0.07	0.54	0.04	0.05
No	1,068	0.52	0.03	REF	0.52	0.03	REF	0.50	0.03	REF
Body mass index										
Normal weight	289	N/A	N/A	N/A	0.53	0.03	REF	0.52	0.04	REF
Overweight	322	N/A	N/A	N/A	0.54	0.04	0.69	0.53	0.04	0.68
Obese grade I	235	N/A	N/A	N/A	0.54	0.04	0.48	0.52	0.03	0.74
Obese grade II	151	N/A	N/A	N/A	0.54	0.03	0.56	0.53	0.04	0.72
Obese grade III	109	N/A	N/A	N/A	0.61	0.04	<0.01	0.59	0.03	<0.01
Underweight	21	N/A	N/A	N/A	0.46	0.05	0.05	0.44	0.05	0.05

BMI classifications: normal weight (BMI 18.5–24.9 kg/m^2^); overweight (BMI 25.0–29.9 kg/m^2^); obese grade I (BMI 30.0–34.9 kg/m^2^); obese II (BMI 35.0–39.9 kg/m^2^); obese III (BMI ≥ 40.0 kg/m^2^); underweight (BMI < 18.5 kg/m^2^).

All models were adjusted for sex, age at assessment, and age at diagnosis.

Nine survivors did not complete the six-minute walk and were removed from analysis due to uncontrolled asthma (1), paralysis (2), painful swelling in legs (2), acute neurological symptoms (1), chronic low back pain (2), and a recent triple bypass surgery (1).

Seven community controls were removed from analysis due to a recently placed pacemaker (1), acute angina (1), technical error (2), severe COPD (1), unable to ambulate without assistance (1), severe shortness of breath (1).

BMI, body mass index; <, less than; n, number; N/A, not applicable; R^2^, Coefficient of determination; REF, reference.

^a^ Additionally adjusted for smoking status and physical activity status.

### Quality of life and adaptive physical function

Table 4 shows associations between the PCI, group (survivors or community controls), PA status, and the PCS and MCS of the SF-36. Survivors had significantly lower MCS and PCS scores than community controls. However, scores were not impacted by the energy cost of walking, in models with or without interaction terms (PCI by group status). Participants who met CDC PA guidelines had higher scores on the MCS and PCS. Results were similar for the other SF-36 subscales. Adaptive physical function was measured with PPT and TUG time (Table 4). Compared to community controls, survivors scored lower on the PPT. There was no association between the PCI, PPT, or SF-36 scores. Survivors with higher PCI scores had significantly longer TUG times compared to community controls with higher PCI scores.

**Table 4:** Associations between the physiological cost index, SF-36 component summaries, and adaptive physical function in adult survivors of acute lymphoblastic leukemia vs. community controls.

	SF-36						Adaptive physical function					
	PCS^a^			MCS^b^			PPT^c^			Timed up and go^a^		
	mean	SE	*p*	mean	SE	*p*	mean	SE	*p*	mean	SE	*p*
PCI	51.70	0.09	0.26	48.41	0.07	0.94	26.91	0.11	0.78	5.86	0.02	0.11
Group												
Community controls	53.47	0.43	REF	48.83	0.53	REF	27.46	0.06	REF	5.36	0.12	REF
Survivors	50.20	0.31	<0.01	46.80	0.38	<0.01	26.59	0.09	<0.01	6.26	0.14	0.82
PA												
Inactive	50.29	0.35	REF	46.78	0.46	REF	26.68	0.07	REF	6.18	0.13	REF
Active	53.43	0.34	<0.01	48.86	0.43	<0.01	27.37	0.08	<0.01	5.43	0.13	<0.01
PCI*group, control	54.71	0.14	REF	49.94	0.12	REF	27.60	0.02	REF	5.19	0.03	REF
PCI*group, survivor	50.46	0.09	0.18	47.62	0.08	0.39	26.63	0.01	0.19	6.14	0.02	<0.01

Physical activity guidelines based on the 2018 CDC recommendations of 450 metabolic equivalent minutes per week.

Interaction term was removed from the model when not significant.

Nine survivors did not complete the six-minute walk and were removed from analysis due to uncontrolled asthma (1), paralysis (2), painful swelling in legs (2), acute neurological symptoms (1), chronic low back pain (2), and a recent triple bypass surgery (1).

Seven community controls were removed from analysis due to a recently placed pacemaker (1), acute angina (1), technical error (2), severe COPD (1), unable to ambulate without assistance (1), severe shortness of breath (1).

42 (sixteen community controls, 26 survivors) did not complete their SF-36 questionnaire.

One survivor did not complete their physical performance test due to cognitive limitations.

Three survivors did not complete timed up and go due to patient refusal (1) and tester error (2).

SF-36, Medical Outcomes Survey Short-Form-36; MCS, mental component summary; PCI, physiological cost index; PCS, physical component summary; PPT, physical performance test.

^a^ Model additionally adjusted for age, sex, physical activity, smoking status, and race.

^b^ Model additionally adjusted for age, sex, physical activity, and smoking status.

^c^ Model additionally adjusted for physical activity, sex, and smoking status.

### Associations between PCI, quality of life, and adaptive physical function

**Figure 1:**
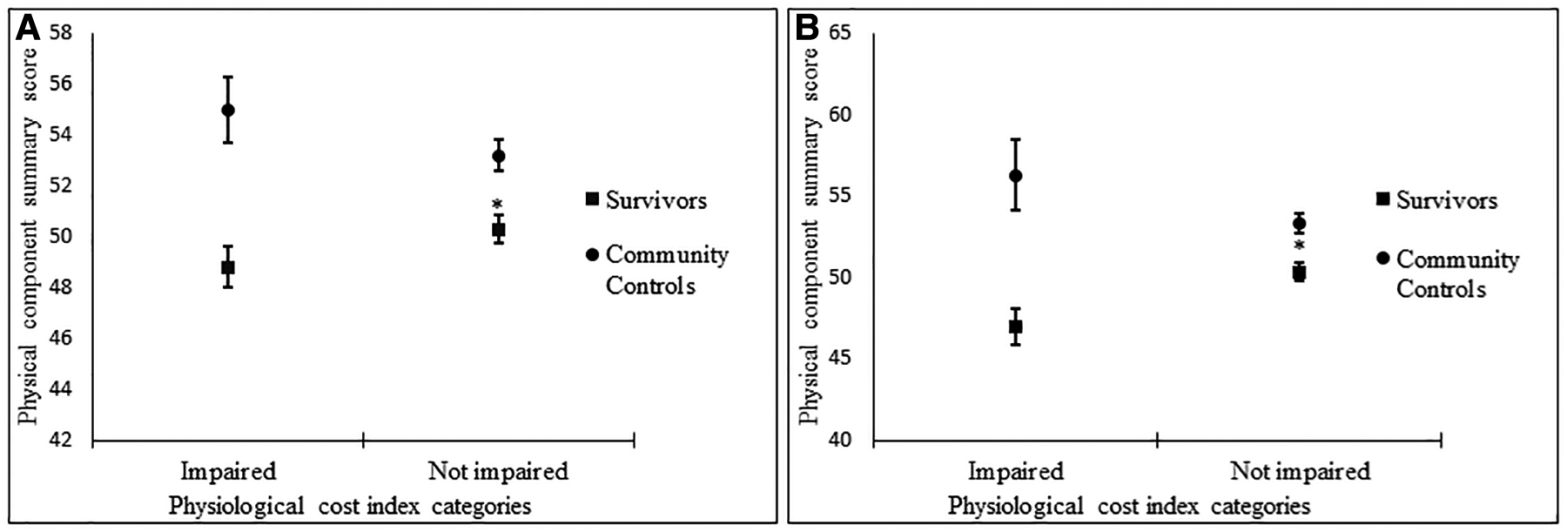
Associations between impaired vs. not impaired physiological cost index and physical component summary score (**A**), severely impaired vs. not impaired physiological cost index, physical component summary score (**B**).

To further assess HRQoL, participants were categorized by PCI as impaired (>90th percentile) and not impaired (<90th percentile), as well as severely impaired (>95th percentile) and not severely impaired (<95th percentile). Impairment was associated with lower PCS scores in survivors but not community controls (Figure 1A). Severe impairment was also associated with lower PCS in survivors but not community controls (Figure 1B).

## Discussion

Adult survivors of childhood ALL who are morbidly obese (BMI ≥ 40 kg/m^2^) have an increased energy cost of walking. This association persists after accounting for treatment exposures and other host factors, including persistent symptoms of motor neuropathy. Moreover, impaired waking efficiency impacts activities of daily life, as survivors who have increased energy cost of walking take longer to complete the TUG, an indicator of general mobility and future functional decline (42). Decreased walking efficiency also impacts perceived well-being among survivors as evident by the lower physical HRQoL reported by survivors with the most impaired energy cost of walking (>90th percentile and >95th percentile) compared to survivors not impaired. Community controls whose energy cost of walking is the most impaired do not report impaired HRQoL, suggesting that survivors, compared to persons without a history of childhood ALL, have more difficulty compensating for obesity during day-to-day activities. Because obesity is potentially modifiable, these data support the need for tailored weight loss interventions that consider the specific energy costs of adult survivors of childhood ALL.

Our finding of increased energy cost of walking among young adult survivors of childhood ALL, compared to community controls, is supported by a study that examined energy cost of walking in younger ALL survivors, but that did not consider obesity or neuropathy as risk factors. Warner et al. compared differences in heart rate at rest and during a treadmill walking test [set speed of 2 kilometers/hour (km/h)] in children and adolescents after treatment for ALL (median age 12.3 years; range 7.2–18.2 years) and sibling controls (43). In their study, heart rate differences were greater among ALL survivors [mean: 112 beats per min (bpm) range: 85–134 bpm] than siblings (mean: 101 bpm; range 75–128 bpm, *p* < 0.01). As stated, the impact of obesity on this association was not examined, however, survivors had higher BMI values [1.27 standard deviation score (SDS)] than siblings (0.62 SDS). Our additional findings that both obesity and peripheral neuropathy are independently associated with energy cost of walking are supported by studies in populations with neuromuscular disease, that identified associations between gait patterns and energy cost of walking, and by studies in the general population, that observed independent effects of obesity and gait kinematics, on energy cost of walking.

Abnormal gait mechanics, predicted by degree of neuromuscular impairment, are associated with the energy cost of walking in studies that include persons with multiple sclerosis (*n* = 33, mean age 41 ± 1.7 years) and those with Charcot Marie Tooth Disease (*n* = 8, median age 34 ± 9.7 years; 37.5% male) (44, 45). Obesity was not included as a risk factor in either of these studies. In studies of the general population, obesity increases the energy cost of walking by 10%–33% (10, 46), independent of abnormal gait biomechanics (47). Adult survivors of childhood ALL are at an increased risk for obesity (3), which confers increased demand, and peripheral motor neuropathy (48, 49), which appears to limit their ability to respond to increased demand (48, 49). Thus, to promote PA in adult survivors of childhood ALL, those who are obese, or have peripheral neuropathy, will need an intervention that addresses both impairments.

In our analysis, when obesity is measured by body fat percentage, rather than BMI, the impact of excess mass on the energy cost of walking is similar between adult ALL survivors and community controls. This suggests that BMI is a measure that does not adequately characterize obesity in adult ALL survivors. As we have previously shown, BMI misclassifies a large proportion of childhood cancer survivors (47% males; 53% females) as obese, when compared to the gold standard (body fat percentage) (24). Alternatively, it may be that body composition is less impactful on energy costs of walking than actual body size among survivors, whose muscle quality may be impaired (50), resulting in altered gait dynamics and reduced gait efficiency. The latter hypothesis is supported by Browning et al. who examined the effects of both excess weight measured by BMI and body composition, measured by dual energy x-ray absorptiometry, on the energy cost of walking at set speeds (0.5, 0.75, 1.00, 1.25, 1.50, or 1.75 m/s) in 39 otherwise healthy persons, some of whom who were obese (mean age 24.5 ± 5.1 years; 48.7% obese; 51.2% male) (51). Participants with obesity (BMI > 30 kg/m^2^) had 10% higher energy cost of walking, whereas body composition only explained a small part of the increase (r^2 ^= 0.15). In another study, Griffin et al. evaluated the impact of adding lead weights to a waist band (0, 10, 20, or 30% of body weight) on energy cost of walking in eight healthy individuals (mean weight 68.7 ± 12.5 kg; mean age 26.0 ± 5.0 years; 50% male) (52). Adding 30% of body weight increased cost of walking by 47 ± 17%. Survivors of childhood ALL also have impaired lower body strength compared to their peers (50), which could further limit their capacity to compensate for excess weight. Hunter et al. reported a significant association between isometric quadriceps strength and energy cost of walking in 66 overweight premenopausal women (mean age 34.6 ± 6.2 years). They showed an increase of 36 newtons of quadriceps force was associated with a lower peak oxygen uptake requirement [VO_2_ (mL/kg/min) *β* −0.37; *p* < 0.01] at a set walking speed of 4.84 km/h (53). Thus, in addition to addressing obesity and neuropathy to increase ease of movement and promote PA in adult survivors of childhood ALL, increasing muscle strength should be further researched as another component to raise walking efficiency.

Although our study is not the first to report lower adaptive physical function (slower TUG time) or lower scores on measures of HRQoL among ALL survivors compared to community controls (50, 54), our data adds to existing literature by documenting that deficits in adaptive physical function and HRQoL are associated with the energy cost of movement in this vulnerable population. Deficits in adaptive physical function and HRQoL are problematic and relevant. In the general adult population (*n* = 200, age range 20–50 years, 44.5% male), slower TUG time is associated with higher scores on the cumulative illness rating scale and with lower scores on the PCS SF-36 Summary Scale (55). Among older adults, increasing time on the TUG is associated with risk of future falls, hospitalizations, and functional decline (56–58). When dichotomized, a decline below 50 on the PCS SF-36 Summary Scale is associated with a 58% (95% CI, 30%–91%) increased risk of mortality in women; (59) and when evaluated as a continuous measure, every three point decrement on the PCS increased risk for mortality by 27% (60). Thus, because increased energy cost of walking from obesity is associated both adaptive physical function and HRQoL, early weight management strategies in survivors of childhood ALL, if successful, are likely to have a significant impact on long term health.

Our study has some important strengths. First, we have a large, well characterized study population with not only detailed clinical data, but also detailed disease, treatment, and lifestyle information. This allows for robust analysis and control of confounders of our estimates. Second, our study has a control group, which allows for direct comparisons to a representative sample of the general population, as well as relevant categorizations of impaired energy cost using control percentile scores. Third, objective measures of predictors (BMI and body fat percentage) and outcomes (adaptive physical functioning) limit measurement bias associated with non-objective measures and self-report.

Our study is, however, not without limitations. These data are cross-sectional: temporal associations between obesity, energy cost of walking, adaptive physical function and HRQoL cannot be definitively determined. For example, in an older population, poor physical function is associated with increasing energy costs performing submaximal activities, and survivors can develop functional deficits during treatment that persist through their childhood. More research should be done to exam the temporality between function and energy costs. In addition, our sample was treated at one institution (St. Jude Children’s Research Hospital) between 1962 and 2012; our data may not be generalizable to adult survivors of ALL treated more recently, or at other institutions with different treatment regimens. Furthermore, not every eligible participant agreed to take part in the study. This may bias our estimates of the association between obesity and energy cost of walking. For example, if non-participants were healthier, and less obese, the direction of the association between obesity and the energy cost of walking would be away from the null. Conversely, if the non-participants were less healthy and more obese, the direction of the association would be towards the null. In our study, while we used a self-report questionnaire widely used in population cohorts to characterize PA (35), these data are prone to error when compared to data collected by doubly labelled water or accelerometry (61, 62). Additionally, using calipers to ascertain body fat percentage increases measurement error, thus increase the risk of misclassification bias. Finally, our study looked at the associations between obesity, neuropathy, and energy costs of walking, however, other factors could be impacting energy costs of walking in our survivors. Some examples include muscle weakness, balance, and compensation for chronic pain (63, 64). Future studies should examine these exposures impact on energy costs of walking.

However, using calipers for body fat percentage is valid replacement for the dual energy x-ray absorptiometry in both the general population and childhood cancer survivors (24, 65). Finally, while the PCI is a valid measure of the energy cost of walking, and easy to do across clinical settings, it is not the gold standard, which would include evaluating energy consumption during walking using a portable metabolic cart.

Nevertheless, this study provides important information about the impact of obesity on daily function in adult survivors of childhood ALL. Among survivors, but not among community controls, obesity increased the energy cost of walking, suggesting that the survivors have difficulty compensating for excess weight. Unfortunately, due to their increased energy cost of walking, survivors have disproportionally worse adaptive physical function and HRQoL, compared to community controls. Tailored interventions, accessible to those with increased energy cost of walking, with a focus on weight loss and decreasing the burden of peripheral neuropathy, are needed for this vulnerable population. Providing adaptive equipment or exercise modifications to help manage neuropathy, accompanied by behavioral strategies that promote weight loss and encourage physical activity, should be considered.

## Data Availability

The data supporting the conclusions of this article will be made available by the authors, without undue reservation.
