# Supplementary material for: Energy cost of walking in obese survivors of acute lymphoblastic leukemia: A report from the St. Jude Lifetime Cohort
Source: Front Pediatr. 2022 Oct 28;10:976012. doi: 10.3389/fped.2022.976012 (PMC9650430; doi:10.3389/fped.2022.976012)
Supplement: Supplementary file 1 [file Table1.docx]

**Supplemental Figure 1. CONSORT diagram**
