# Supplementary material for: Energy cost of walking in obese survivors of acute lymphoblastic leukemia: A report from the St. Jude Lifetime Cohort
Source: Front Pediatr. 2022 Oct 28;10:976012. doi: 10.3389/fped.2022.976012 (PMC9650430; doi:10.3389/fped.2022.976012)
Supplement: Supplementary file 2 [file Table2.docx]

| Supplemental Table 1. Characteristics of adult survivors of childhood acute lymphoblastic leukemia, participants vs. non-participants | | | | | | |
| --- | --- | --- | --- | --- | --- | --- |
|  | Participants  (n=1,166) | | Non-Participants  (n=355) | |  | |
|  |  |  |  |  | p | |
| Age at evaluation in years, mean (SD) | 37.3 | (9.9) | 34.8 | (10.1) | <0.01 | |
| Age at diagnosis in years, median (range)^a^ | 5.3 | (5.3-21.0) | 6.4 | (4.6-20.1) | 0.06 | |
| Sex, n (%) |  |  |  |  |  |  |
| Male | 607 | (52.0) | 190 | (53.5) | 0.62 | |
| Female | 559 | (48.0) | 165 | (46.5) |  |  |
| Race, n (%) |  |  |  |  |  |  |
| White | 1,036 | (88.8) | 303 | (85.4) | 0.20 | |
| Black | 104 | (8.9) | 42 | (11.8) |  |  |
| Other | 26 | (2.2) | 10 | (2.8) |  |  |
| **Glucocorticoids** |  |  |  |  |  |  |
| Prednisone, n (%) |  |  |  |  |  | |
| Yes | 1153 | (98.9) | 345 | (97.2) | 0.02 | |
| No | 13 | (1.1) | 10 | (2.8) |  |  |
| Hydrocortisone, n (%) |  |  |  |  |  |  |
| Yes | 783 | (67.2) | 181 | (51.0) | <0.01 | |
| No | 383 | (32.8) | 174 | (49.0) |  |  |
| Dexamethasone, n (%) |  |  |  |  |  |  |
| Yes | 375 | (32.2) | 68 | (19.2) | <0.01 | |
| No | 791 | (67.8) | 287 | (80.8) |  |  |
| **Antimetabolites** |  |  |  |  |  |  |
| Methotrexate, n (%)^b^ |  |  |  |  |  |  |
| Yes | 1166 | (100) | 353 | (99.4) | 0.06 | |
| No | 0 | (0) | 2 | (0.2) |  |  |
| 6-Mercaptopurine, n (%) |  |  |  |  |  |  |
| Yes | 1152 | (98.8) | 340 | (95.8) | <0.01 | |
| No | 14 | (1.2) | 15 | (4.2) |  |  |
| Cytarabine, n (%) |  |  |  |  |  |  |
| Yes | 960 | (82.3) | 267 | (75.2) | <0.01 | |
| No | 206 | (17.7) | 88 | (24.8) |  |  |
| **Vinca Alkaloids** |  |  |  |  |  |  |
| Vincristine, n (%)^b^ |  |  |  |  |  |  |
| Yes | 1165 | (99.1) | 353 | (99.4) | 0.12 | |
| No | 1 | (0.09) | 2 | (0.6) |  |  |
| **Anthracyclines** |  |  |  |  |  |  |
| Doxorubicin, n (%) |  |  |  |  |  |  |
| Yes | 278 | (23.8) | 64 | (18.1) | 0.02 | |
| No | 888 | (76.2) | 291 | (81.9) |  |  |
| Daunorubicin, n (%) |  |  |  |  |  |  |
| Yes | 808 | (69.3) | 214 | (60.3) | <0.01 | |
| No | 358 | (30.7) | 141 | (39.7) |  |  |
| **Epipodophyllotoxins** |  |  |  |  |  |  |
| Teniposide, n (%) |  |  |  |  |  |  |
| Yes | 470 | (40.3) | 144 | (40.6) | 0.93 | |
| No | 696 | (59.7) | 211 | (59.4) |  | |
| Etoposide, n (%) |  |  |  |  |  |  |
| Yes | 550 | (47.2) | 137 | (38.6) | <0.01 | |
| No | 616 | (52.8) | 218 | (61.4) |  |  |
| **Asparaginase** |  |  |  |  |  |  |
| L-Asparaginase, n (%) |  |  |  |  |  |  |
| Yes | 1053 | (90.3) | 310 | (87.3) | 0.11 | |
| No | 113 | (9.7) | 45 | (12.7) |  |  |
| Erwinia, n (%) |  |  |  |  |  |  |
| Yes | 156 | (13.4) | 34 | (9.6) | 0.06 | |
| No | 1010 | (86.6) | 321 | (90.4) |  |  |
| **Cranial radiation,** n (%) |  |  |  |  |  |  |
| Yes | 556 | (47.7) | 193 | (54.4) | 0.03 | |
| No | 610 | (52.3) | 162 | (45.6) |  |  |
| ^a^Wilcox on Rank Sum test used for analysis  ^b^Fisher Exact test used for analysis  <, less than; n, number; %, percent; p, probability; SD, standard deviation | | | | | | |
