# Supplementary material for: Energy cost of walking in obese survivors of acute lymphoblastic leukemia: A report from the St. Jude Lifetime Cohort
Source: Front Pediatr. 2022 Oct 28;10:976012. doi: 10.3389/fped.2022.976012 (PMC9650430; doi:10.3389/fped.2022.976012)
Supplement: Supplementary file 3 [file Table3.docx]

| Supplemental Table 2. Associations between body mass index, body fat percentage, and physiological cost index in adult survivors of childhood acute lymphoblastic leukemia treated after 1980 vs. community controls | | | | | | | | | |
| --- | --- | --- | --- | --- | --- | --- | --- | --- | --- |
|  |  |  | Physiological cost index (BMI model) | | |  | Physiological cost index (Body fat percentage model) | | |
|  | n |  | mean | SE | p |  | mean | SE | p |
| Group |  |  |  |  |  |  |  |  |  |
| Control | 484 |  | 0.49 | 0.010 | REF |  | 0.49 | 0.008 | REF |
| Survivor | 902 |  | 0.51 | 0.009 | 0.05 |  | 0.50 | 0.007 | 0.16 |
| Body mass index |  |  |  |  |  |  |  |  |  |
| Normal weight | 437 |  | 0.49 | 0.009 | REF |  | N/A | N/A | N/A |
| Overweight | 391 |  | 0.49 | 0.009 | 0.97 |  | N/A | N/A | N/A |
| Obese grade I | 262 |  | 0.50 | 0.011 | 0.47 |  | N/A | N/A | N/A |
| Obese grade II | 160 |  | 0.50 | 0.013 | 0.37 |  | N/A | N/A | N/A |
| Obese grade III | 112 |  | 0.55 | 0.009 | <0.01 |  | N/A | N/A | N/a |
| Underweight | 31 |  | 0.46 | 0.029 | 0.32 |  | N/A | N/A | N/A |
| Body fat percentage |  |  |  |  |  |  |  |  |  |
| Normal | 785 |  | N/A | N/A | N/A |  | 0.48 | 0.008 | REF |
| Excess | 608 |  | N/A | N/A | N/A |  | 0.51 | 0.007 | <0.01 |
| Note: BMI classifications: normal weight (BMI 18.5-24.9 kg/m^2^); overweight (BMI 25.0-29.9 kg/m^2^); obese grade I (BMI 30.0-34.9 kg/m^2^); obese II (BMI 35.0-39.9 kg/m^2^); obese III (BMI ≥40.0 kg/m^2^); underweight (BMI <18.5 kg/m^2^).  Normal body fat percentage (males: <27.5%; females: <39.9%); excess body fat percentage (males: ≥27.5%; females: ≥39.9).  Models were adjusted for sex, age at evaluation, physical activity status, height^2^ and smoking status  BMI, body mass index; n, number; N/A, not applicable; %, percent; p, probability; SE, standard error; REF, reference | | | | | | | | | |
